# Proceedings of the Mississippi Valley Dental Association

**Published:** 1868-07

**Authors:** 


### Proceedings of Societies.

### PROCEEDINGS OF THE MISSISSIPPI VALLEY DENTAL ASSOCIATION.

The twenty-fourth annual meeting of the Mississippi Valley Dental Association met in the lecture room of the Ohio Dental College, March 4th, 1868, President W. H. Goddard in the chair ; G. W. Field, Secretary.

Members present: Drs. A. A. Blount, J. G. Cameron, W. H. Shadoan, J. Taft, W. H. Morgan. G. W. Field, M. Wells, S. P. Cutler, W. T. Arrington, Geo. H. Cushing, W. M. Hammond, M. DeCamp, R. L. Evans, M. M. Oldham, H. A. Smith, G. W. Keely, N. W. Williams, J. A. McClelland, G. W. Baxter, A. Berry, H. McCullum, H. A. Beamer.

Minutes of last session were read.

The Executive committee presented the following report which was adopted :

### ORDER OF BUSINESS.

1. Report of officers.
2. Election of officers. 2 P. M. first day.
3. Miscellaneous business.

Report of committees in order at any interval of discussion.

4. Essays or papers to be read on the opening of discussion.

### SUBJECTS FOR DISCUSSION.

1. Means for controlling the flow of saliva.
2. Preparation of cavities for filling.
3. Filling—methods and materials.
4. Dental quackery—its remedy.

5. Mechanical Dentistry.
6. Exhibition of instruments and appliances.

G. W. KEELY,

W. G. REDMAN, } *Committee.*

D. R. JENNINGS,

It was moved and adopted that the President appoint a committee on Nominations.

The following committee was therefore appointed: Drs. H. A. Smith, W. H. Shadoan, and A. A. Blount.

Dr. A. Berry being the only member present of the Membership committee. The vacancies were filled by the following appointments by the chair, Drs. Keely, and*

They reported Drs. W. T. Arrington, of Memphis, Tenn., and S. P. Cutler, of Holly Springs, Miss., as eligible to membership, both of whom were unanimously elected.

On motion it was resolved that 7½ o'clock, Thursday evening, be set for a lecture on Nitrous Oxide by Dr. George Watt.

Certain matters pertaining to professional ethics were here referred to the Executive committee.

The Membership committee presented names for admission as follows:

Drs. Wm. E. Tate, New London, Conn.; W. H. Thrift, Boonsboro, Iowa; R. J. Omahoney, Lexington, Ky.; J. P. Holmes, Terry, Hinds Co., Miss.; B. F. Mills, Brooklyn, N. Y.; R. N. Lawrence, Atlanta, Ill.; D. C. Whaley, Pomeroy, O.; W. A. Graham, Madison, Ind. All elected.

The hours for the daily sessions were set as follows: 9 to 12 A. M. 2 to 5 P. M. 7½ to 10 P. M.

Adjourned until 2 P. M.

AFTERNOON SESSION.

Regular business suspended.

The following motion was offered by Dr. J. Taft:

*Resolved*, That the Chair appoint a committee of two to

*It does not appear who the other was. (Sec.)

examine instruments and appliances, and that Thursday 2, P. M. be a special hour for their report.

The Association went into an election for officers which resulted as follows :

*President*, G. H. Cushing, Chicago, Ill.
*Vice President*, W. T. Arrington, Memphis, Tenn.
*Recording Secretary*, Wm. Taft, Cincinnati, O.
*Corresponding Secretary*, M. Wells, Indianapolis, Ind.
*Treasurer*, J. G. Cameron, Cincinnati, O.

Drs. Blount and DeCamp were appointed to conduct the President elect to the chair, after which the retiring President delivered his address.

On motion,

*Resolved*, That a vote of thanks be tendered retiring officers for the faithful performance of their duties.

On motion, the Association endorsed the sentiments contained in the address, and requested a copy for publication.

Dr. A. Berry, chairman of the committee appointed in '67 to raise funds for entertaining the American Dental Association, last summer, presented his report which was accepted.

The committee appointed to procure a reporter suggested that Dr. J. Taft be appointed to report for the Society, which was adopted and the committee discharged.

The first subject for discussion, on the programme, was announced and taken up.

*Resolved*, That to-morrow morning, 10 o'clock, be appointed a special hour to enable members to practically illustrate their methods of applying instruments for controlling saliva. Passed.

Adjourned until Thursday morning, 9 A. M.

### THURSDAY—MORNING SESSION.

Association called to order. President Cushing in the chair.

On motion, Dr. Geo. E. Hayes, of Buffalo, was elected an honorary member of the Association.

On motion, Dr. G. W. Keely was excused from the committee on Instruments and Appliances. Dr. N. W. Williams was appointed in his stead.

The following communication from Dr. B. D. Wheeler, ex-treasurer was read.

GENTLEMEN:—Having been elected by you Treasurer, at the last annual meeting, I would respectfully state that the books and funds of the Association have not been in my hands until last evening, hence I have made no report until now. I know nothing of the books farther than the former treasurer reports to me one hundred and ninety-one dollars on hand, for which I hold his check. I would suggest the appointment of an Auditing committee, when I will be prepared to hand over the books to the treasurer elect.

Respectfully,

B. D. WHEELER.

Drs. Morgan and H. A. Smith were appointed to audit the Treasurer's accounts.

On motion it was agreed to donate fifty dollars ($50) to the Ohio Dental College for the purpose of fitting up the room which we now occupy.

Dr. Cutler was called upon for an essay, which was read.

*Resolved*. That the thanks of this Association be tendered Prof. Cutler for his able and suggestive paper, and that a copy be requested for publication.

The Auditing committee presented the following report:

*To the Mississippi Valley Dental Association:*

The Auditing committee appointed by you to examine the books and vouchers of the Treasurer of this Association would respectfully report that they have performed the duty and find the amount of balance in the hands of the former treasurer is $239, 35-100, instead of the amount first named. That amount ($239, 35-100 is now in the hands of the treasurer of last year. The mistake occurred in the hurry of making up the report of the treasurer of 1866—67.

W. H. MORGAN, } Committee.

H. A. SMITH, }

Report accepted.

The committee on Prize Essays reported as follows:

The committee respectfully report that an essay on the subject of Alveolar Abscess, and one on Disease of the Antrum have been presented for our consideration.

Both of these give evidence of labor and care in their preparation, yet we do not find them in advance of the current literature of our profession on the same subjects, and we therefore think, that notwithstanding their merits, they are not entitled to the offered rewards.

GEO. WATT, }

G. W. KEELY, } *Majority of Com.*

H. A. SMITH, }

C. M. WRIGHT, }

On motion, it was

*Resolved*, That a committee on Ethics be appointed by the President to serve for the ensuing year.

Also, it was

*Resolved*, That the committee appointed to examine instruments and appliances be requested to bring in all instruments and appliances in their possession for exhibition at 2 P. M., and exhibit, describe and answer all questions in reference to them.

Dr. H. A. Smith notified the Association that he had a paper prepared, and would present it to the society if it was their pleasure. Whereupon he was requested to read it. Received, and a copy asked for publication. The paper was very interesting and called forth considerable discussion.

AFTERNOON SESSION.

Association called to order. Vice-President W. T. Arrington in the chair.

The hour having arrived for the report of the committee on Appliances, &c., it was presented as follows:

Your committee appointed to examine new appliances, &c. have attended to that duty, and beg leave to submit the following report:

The annealing lamp presented by Dr. Hayes, of Buffalo, is the most complete and convenient in use.

The Hayes packing flask is the most complete apparatus ever examined by the committee.

The Hayes steam compressor, for closing the flask while vulcanizing, your committee deem one of the most ingenious and complete apparatus of the kind ever presented to the profession, although somewhat complicated it is readily understood, and not only saves time but packs perfectly without the liability of breaking the thinnest gum. We take great pleasure in recommending it to your favorable notice, and at the same time express our thanks to Dr. Hayes for his indefatigable labors in perfecting Dental apparatus.

The articulator presented by Dr. Hayes is perfect in all its parts, and combines all that is required in an articulator.

Dr. Hammond, of Cincinnati, presented a napkin holder, very simple, and well adapted to its use.

Dr. McClelland, of Louisville, presented specimens of rose pearl base. From the very superficial examination had of it by your committee, they are unprepared to give a decided opinion, but believe from the representations made by the Doctor and others, that it is worthy of the careful attention and scrutiny of the profession, and we hope that it may supercede rubber in artificial dentures. We are assured that the secretions of the mouth will not affect it in the slightest degree, and that it is proof against the most powerful acids, nor is not acted upon by oils. It is also light, strong, and flexible, and is more readily repaired than any other artificial base.

M. DeCamp,<br>

A. A. Blount,<br>

N. W. Williams, } *Committee.*

Dr. Hayes exhibited and explained the instruments laid before the society by him.

On motion, it was

*Resolved*, That an invitation be extended to Prof. Cutler and Arrington to remain through the meeting of the Association, and that Prof. Cutler be invited to deliver a lecture upon microscopy.

*Resolved*, That the committee on Ethics be instructed to adopt for their government the seventh by-law of the Ohio State Dental Society, as follows:

All complaints against members for violating the Constitution and By-Laws, gross immorality or unprofessional conduct, shall be presented in writing to the committee on Ethics, who shall proceed to furnish the accused a copy of the complaint. Call both him and accuser before them. Examine and decide on the case, and report their decision to the next regular meeting of the society.

Dr. McClelland presented and described his new base for artificial teeth, called rose pearl.

A number of instruments, presented too late for consideration in the committee's report, consisting of excavators and pluggers, manufactured by S. S. White, of the most approved and favorite patterns of the most eminent operators in the United States, were laid before the Association for their examination. They were exquisitely finished, excellently tempered, and with the perfectness of the points well calculated to inspire with enthusiasm all who appreciate the necessity of such instruments.

Professors Cutler and Arrington signified their acceptance of the invitation to remain during the sessions of this meeting. 8½ o'clock this evening was the hour appointed for the lecture of Dr. Cutler on the microscope.

On motion, it was resolved to take up the postponed discussion of arsenic, and its effects upon the teeth.

Adjourned to meet this evening at 7½ o'clock.

THURSDAY EVENING.

The Association met at the appointed hour.

The evening was occupied by the very interesting lectures of Drs. Geo. Watt, on the subject of Nitrous Oxide Gas, and S. P. Cutler, on Microscopy.

FRIDAY—MORNING SESSION.

Association called to order. President in the chair.

Dr. Goddard, Secretary *pro tem.*

On motion, Dr. W. H. Goddard, and A. Berry were appointed a committee to nominate delegates to the American Dental Association, meeting July 28th, next.

The President appointed the following committee on Ethics: Drs. Geo. Keely, J. Taft, and W. H. Morgan.

The committee to appoint delegates to represent this body in the American Dental Association present the following names for confirmation:

Drs. R. L. Evans, Toledo, O.; W. E. Tate, New London, Conn.; W. H. Thrift, Boonesboro, Iowa; R. J. Omahoney, Lexington, Ky.; J. P. Holmes, Terry, Miss.; B. F. Mills, Champaign, Ill.; R. E. Lawrence, Atlanta, Ill; D. C. Whaley, Pomeroy, O.; W. A. Graham, Madison, Ind.; S. Clippinger; W. M. Hammond, Cincinnati, O.

A. Berry, } *Committee.*

W. H. Goddard, }

Printing bill was ordered to be paid.

Reporter's bill was ordered to be paid.

Janitor ordered paid $15.

*Resolved*, That the chair appoint a committee to make new appointments to fill the various committees of the Association, made vacant by expiration of term.

Drs. J. A. McClelland and J. G. Cameron, were appointed.

The committee reported the following appointments:

*Executive Committee.*—Drs. S. P. Cutler, H. A. Smith, and A. A. Blount.

*Publishing Committee.*—Drs. J. Taft, C. M. Wright, and A. Berry.

*Committee on Membership.*—Drs. S. Driggs, N. W. Williams, and D. C. Whaley.

Dr. Berry offered the following:

*Resolved*, That the thanks of this Association be extended to Prof. Cutler for his very instructive lecture, and exhibition with his powerful microscope, of the ramifications of the nerves in the pulp and dentine.

The second subject was taken up and discussed by Drs. Taft, Driggs and Smith.

Report of special committee, on prize essay, on the Health of Dentists.

Your committee respectfully report that but one essay has been presented for their consideration. This they have carefully examined, and find that though in many respects meritorious, it falls short of the qualities which would entitle it to the offered prize of two hundred dollars. Accordingly, we recommend that it be returned to its author.

GEO. WATT,<br>

S. DRIGGS, } *Majority of Committee.*<br>

G. W. KEELY,

The committee appointed to revise the membership roll, reported that they had not been able to perform the duty, and asked to be discharged, and another committee appointed in their stead.

On motion, Drs. Cameron and Wright were appointed committee on Membership Revision.

The third subject taken up for discussion.

Report of the committee on Ethics.

Your committee, to whom was referred the subjects of ethics, would respectfully report, that they have given the matter the most serious consideration, under a sense of its grave importance both as to its moral aspect, and in its relation to the highest interest of this Association and the profession at large. Your committee have had presented to their notice, two advertisements and posters of a most unprofessional character, calculated to degrade not only the individuals putting them forth, but the profession they are bound to honor and elevate. And it is with the gravest sorrow that your committee have received testimony of such unprofessional conduct on the part of members of this Association, one of whom has held the highest position of honor in this society. And your committee feel that the time has come when a decided position should be taken upon this subject, and that such action could come no more appropriately from any other body than this, the oldest Dental society in existence, whose record heretofore has been one we have never felt ashamed to refer to. By every consideration of regard for the interests of the profession, by every principle of morality, and by the code adopted by this body, we are constrained to take a stand both dignified and firm. We wish our trumpet to give no uncertain sound, but in tempering justice with mercy, should desire that the action taken by the society now, may have the effect to bring back into its bosom those whom your committee feel have erred through inadvertance, rather than with malice aforethought.

That our action hereafter, as in the past be had with a view to elevate and advance our professional standard, therefore

*Resolved*, That at the next annual session of this Association, prompt and decisive action shall be taken to expel such members as may be acting in contravention of the code of ethics.

G. W. Keely,<br>

W. H. Morgan, } *Committee.*<br>

Geo. H. Cushing,

The following was presented by Dr. W. T. Arrington :

Whereas, Dental education and associated effort are the only true means of elevating the standard of our profession to that dignified position, side by side with the other learned professions of the day, thereby wresting it from the hands of those ignorant pretenders who shamefully abuse and bring disgrace upon it.

*Resolved*, That no member of this Association shall take into his office a student to be instructed in the science and art of Dentistry, for a less period than two years, the student giving a bond of honor, with good security, that he will never practice Dentistry except in the capacity of a student, until he has received the degree of D. D. S. from a respectable chartered college.

### AFTERNOON SESSION.

Met at the regular hour. Dr. Arrington in the chair.

Fourth subject taken up.

On motion of Dr. J. Taft, it was decided to appoint a committee to revise the Constitution and By-laws that are scattering, and report at the next annual meeting. Drs. J. Taft, and Geo. Watt appointed by the chair.

Discussion continued.

On motion, it was resolved that a committee be appointed to prepare as soon as possible an essay for distribution to the profession, at cost, for popular reading.

Drs. C. M. Wright, J. Taft and H. A. Smith were appointed.

On motion, the Association adjourned to meet the first Wednesday in March, 1869, in the lecture room of the Ohio Dental College.

